# Epigenetic regulators are preferentially coordinated with protocadherin gene expression across the human brain: a genome-wide co-expression analysis

**DOI:** 10.3389/fgene.2026.1807347

**Published:** 2026-07-09

**Authors:** Drake H. Harbert

**Affiliations:** Inner Architecture LLC, Canton, OH, United States

**Keywords:** cell-type deconvolution, chromatin remodeling, co-expression, epigenetic regulation, GTEx, human brain, neurodevelopmental disorders, protocadherin

## Abstract

How does the brain build and maintain the precise wiring patterns that distinguish one neuron from another? Clustered protocadherins (PCDHs)—a family of cell-surface molecules that give each neuron a unique identity tag—are central to this process. Their expression is famously controlled by an elaborate locus-specific epigenetic system involving DNA methylation, CTCF binding, and chromatin looping. Whether the activity of the broader epigenetic regulatory machinery is coordinated with protocadherin expression across the human brain has not been systematically tested. Here we show that epigenetic regulators are preferentially co-expressed with protocadherins across multiple human brain regions, suggesting a broader transcriptional coordination than the locus-specific mechanisms previously characterized. Using GTEx v8 RNA-seq data from 2,642 brain samples across 13 regions, we conducted a genome-wide co-expression screen and observed a 6.5‐fold enrichment of epigenetic regulators in the top 5% of PCDH-coordinated genes in prefrontal cortex (Fisher’s exact p = 2.8 × 10^−10^). The enrichment replicated independently across additional brain regions, persisted under multiple sensitivity analyses, was preserved after adjustment for cell-type composition, and replicated in an independent brain-bank cohort. The top-ranked epigenetic regulators converge on a defined set of chromatin-remodeling genes implicated in well-characterized neurodevelopmental syndromes. These findings reframe protocadherin biology by extending its epigenetically coordinated context beyond the locus itself to a broader transcriptional program shared with the chromatin-remodeling machinery associated with neurodevelopmental disease. The conceptual advance is consistent with—though does not by itself establish—direct co-regulation, and identifies a specific set of testable mechanistic hypotheses for how disrupted chromatin-remodeling activity in neurodevelopmental disorders may propagate to PCDH-dependent neuronal identity programs in the human brain.

## 1 Introduction

The clustered protocadherin (PCDH) gene family encodes approximately 50 cadherin-related cell-surface proteins organized in three tandem gene clusters—PCDHA, PCDHB, and PCDHG—on human chromosome 5q31 (Wu and Maniatis, 1999; Tasic et al., 2002). Individual neurons express distinct, stochastically selected subsets of protocadherin isoforms, generating a cell-surface molecular barcode that underlies neuronal self-recognition, dendritic self-avoidance, and synaptic specificity (Lefebvre et al., 2012; Mountoufaris et al., 2017; Ing-Esteves et al., 2018). Loss-of-function studies in mice demonstrate that disruption of protocadherin diversity results in severe deficits in dendritic arborization, cortical columnar organization, and neural circuit assembly (Katori et al., 2009; Chen et al., 2017).

The mechanism governing stochastic protocadherin isoform selection is epigenetic. Each clustered protocadherin gene possesses its own promoter, and promoter choice is regulated by differential DNA methylation: active promoters are hypomethylated while silenced promoters are hypermethylated (Kawaguchi et al., 2008; Toyoda et al., 2014). CTCF (CCCTC-binding factor) binds to unmethylated promoter regions and, together with the cohesin complex, mediates long-range chromatin looping between selected promoters and a downstream enhancer element (termed the “super-enhancer” or HS5-1 region), thereby activating transcription of the selected isoform subset (Guo et al., 2012; Monahan et al., 2012; Canzio et al., 2019). This regulatory architecture makes the clustered PCDH locus one of the most complex epigenetically governed regions in the mammalian genome.

While the molecular mechanisms of protocadherin regulation have been extensively characterized in cell lines and mouse models, it remains unknown whether inter-individual variation in the expression of epigenetic regulators—including chromatin remodelers, histone modifiers, and DNA methyltransferases—covaries with protocadherin expression levels across individuals in human post-mortem brain tissue. If such co-expression associations exist, they would be consistent with the regulatory relationship between epigenetic machinery and protocadherin locus control extending to population-level expression variation in the human brain. The present study is designed as a hypothesis-generating co-expression analysis; all findings are correlational and require experimental validation to establish mechanistic relationships or directionality.

This question has particular relevance for neurodevelopmental biology. Loss-of-function mutations in individual epigenetic regulators—including ARID1A, EP300, CREBBP, CHD8, MECP2, KMT2A, KMT2D, and NSD1—cause distinct neurodevelopmental syndromes characterized by intellectual disability, autism spectrum features, or both (Tsurusaki et al., 2012; Roelfsema et al., 2005; Bernier et al., 2014; Amir et al., 1999; Ng et al., 2010; Tatton-Brown et al., 2014; Jones et al., 2012; Kurotaki et al., 2002). Despite affecting different enzymatic activities (chromatin remodeling, histone acetylation, histone methylation, methyl-CpG binding), these mutations converge on overlapping neurodevelopmental phenotypes. Whether protocadherin network disruption represents a common downstream correlate of these diverse epigenetic perturbations has been proposed but not systematically tested at the population level (Hirabayashi and Gotoh, 2010; Mountoufaris et al., 2018).

Here, we applied an unbiased genome-wide co-expression analysis to GTEx v8 RNA-seq data from 2,642 human brain samples across 13 brain regions. Rather than testing predetermined hypotheses, we ranked all 16,225 brain-expressed genes by their coordination with 77 protocadherin family members and assessed whether epigenetic regulators were enriched among the most highly coordinated genes. We replicated the enrichment test independently in three additional brain regions, performed cell-type deconvolution to address the principal confound of bulk RNA-seq co-expression analysis, independently replicated findings in a second dataset (GSE80655), tested condition-specificity across psychiatric diagnoses, confirmed tissue enrichment using peripheral blood data, integrated independent brain DNA methylation evidence, and conducted threshold sensitivity analyses including preranked GSEA.

## 2 Materials and methods

### 2.1 Data sources

Primary analysis used GTEx v8 RNA-seq data (GTEx Consortium, 2020) across 13 brain regions (n = 139–255 per region; total n = 2,642 brain samples). Cross-dataset replication used GSE80655 (Lanz et al., 2019; Brain Bank of the Stanley Medical Research Institute) comprising 281 samples across three brain regions. Whole-blood and non-brain tissue comparisons used GTEx v8 across 41 non-brain tissues (n = 755 for blood). DNA methylation analysis used GSE131706 (Jaffe et al., 2016) BA9 prefrontal cortex methylation array data. Protocadherin family members (n = 77) were defined according to the HUGO Gene Nomenclature Committee PCDH family classification. Epigenetic regulators (n = 52 expressed in BA9; n = 54 total in our curated set) were defined by membership in the GO term “chromatin organization” (GO:0006325) restricted to genes annotated for chromatin remodeling, histone modification, DNA methylation, or methyl-CpG binding.

### 2.2 Co-expression and enrichment analysis

Within-region pairwise Pearson correlations between protocadherin family members expressed at median TPM ≥ 1 were computed for each brain region. FDR-significance was assessed using Benjamini–Hochberg correction at α = 0.05 across all pairs within region. For the genome-wide co-expression screen, the mean Pearson correlation between each brain-expressed gene g (median TPM ≥ 1.0; n = 16,225 in BA9) and all expressed protocadherin family members was computed and used to rank-order all genes by their PCDH-coordination score. The top 5% of ranked genes was designated the primary PCDH-coordinated gene set. The screen was conducted independently in BA9, putamen, hippocampus, and nucleus accumbens.

The curated 52-gene epigenetic regulator set was tested for enrichment in the top 5% of PCDH-coordinated genes using Fisher’s exact test (2-sided). Odds ratios and 95% confidence intervals are reported. Significance threshold was set at α = 0.001 in line with multi-region testing. Enrichment statistics were also computed at top 1%, 5%, and 10% thresholds across all four primary analysis regions to assess robustness to threshold choice. Threshold-free enrichment was assessed using preranked gene set enrichment analysis (GSEA) (Subramanian et al., 2005) on the genome-wide ranking of PCDH-coordination scores, with the 52-gene epigenetic regulator set as the test gene set. Normalized enrichment scores (NES), nominal p-values, and FDR were obtained from 1,000 permutations of the gene set. Functional categories within the epigenetic regulator set were tabulated for the top-ranked genes.

### 2.3 Replication and sensitivity analyses

The genome-wide screen and Fisher’s exact enrichment test were repeated independently in putamen, hippocampus, and nucleus accumbens with no parameters inherited from BA9. Cross-region rank consistency was assessed by pairwise Spearman rank correlation of epigenetic regulator rankings. Subject-level bootstrap (1,000 iterations) was applied to each region to obtain 95% confidence intervals on the mean within-region PCDH correlation. To assess RNA-quality effects, samples were stratified by RNA Integrity Number (RIN) quartile within BA9 and within-region protocadherin co-expression was compared across quartiles.

GSE80655 served as an independent replication cohort. Within-region PCDH co-expression was computed for control samples in anterior cingulate, nucleus accumbens, and DLPFC, and cross-dataset validation of individual gene-pair effect sizes was conducted by correlating GTEx-derived and GSE80655-derived gene-pair correlations. Within GSE80655, control samples were also compared to bipolar disorder, major depressive disorder, and schizophrenia samples to assess the preservation of protocadherin co-expression patterns across psychiatric conditions, with mean change in correlation (Δr) computed per condition–region cell and FDR-significance assessed across the nine total comparisons. Tissue enrichment analysis compared brain PCDH co-expression to non-brain tissue co-expression in GTEx whole blood and 41 additional non-brain tissues. To assess specificity to clustered protocadherins, the analysis was repeated restricted to the 28 clustered PCDHs (Pcdh-α, Pcdh-β, Pcdh-γ).

### 2.4 Confound adjustment

Cell-type proportions were estimated from BA9 expression data via non-negative least-squares (NNLS) deconvolution (Lawson and Hanson, 1974) against a 56-gene marker panel covering six brain cell types: neurons, astrocytes, oligodendrocytes, microglia, endothelial cells, and oligodendrocyte precursor cells (Newman et al., 2015; Kelley et al., 2018; Darmanis et al., 2015; Hodge et al., 2019). Estimated neuron proportions were validated against canonical neuron markers SYN1, SNAP25, and RBFOX3. Cell-type-adjusted PCDH-coordination scores were obtained by partial correlation regressing out the six cell-type proportion variables, after which the genome-wide screen and enrichment test were repeated using adjusted scores. Donor age, sex, and RIN were extracted from GTEx subject metadata; linear regression was applied to residualize expression values against these covariates prior to recomputation of the PCDH-coordination score.

### 2.5 Methylation-level analysis

DNA methylation analysis used GSE131706 (Jaffe et al., 2016) BA9 prefrontal cortex methylation array data to identify CpG probes mapping to PCDH and DNMT genes and to test for coordinated methylation patterns concordant with the transcriptional findings. Coordinated methylation was assessed by computing pairwise correlations between methylation β-values at PCDH and DNMT-mapped CpG probes and comparing their distribution to a permuted null.

### 2.6 Analytical parameters and software

All analyses use median TPM ≥ 1 as the expression threshold for inclusion, Pearson correlation as the primary similarity measure, top-5% as the primary enrichment threshold, and Fisher’s exact test as the primary statistical test. Random seed 42 was used throughout, with FORCE_RECOMPUTE = True to prevent cached intermediate results from masking changes to upstream computation. All analyses were performed in Python 3.10 using NumPy, pandas, SciPy, statsmodels, and matplotlib. GSEA was conducted using gseapy. Source code is available at https://github.com/nwharbert8-ui/epigenetic-pcdh-coordination.

## 3 Results

### 3.1 Protocadherin co-expression is a consistent feature of human brain regions

**FIGURE 1:**
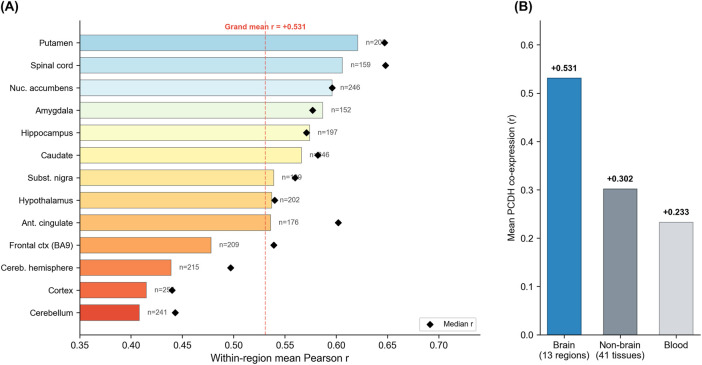
Protocadherin co-expression is strong, positive, and brain-specific across human brain regions. **(A)** Mean within-region Pearson correlation coefficient (r) of all expressed protocadherin gene pairs across 13 GTEx brain regions, ranked from highest (Nucleus accumbens) to lowest (Cortex). The grand mean r = +0.497 across brain regions is indicated by the dashed red line. **(B)** Mean PCDH co-expression compared across tissue categories: 13 brain regions (r = +0.497), 41 non-brain tissues, and whole blood, demonstrating brain-enrichment of the coordinated protocadherin co-expression signal.

Within-region pairwise Pearson correlations between expressed protocadherin family members were strongly positive across all 13 GTEx brain regions (Figure 1; Table 1). The grand mean across regions was r = +0.497 (region-level range +0.355 to +0.595), with subject-level bootstrap 95% confidence intervals excluding zero in every region. Among the four primary analysis regions, FDR-significance rates ranged from 99.7% to 100% of all pairwise PCDH correlations (Table 1). Median within-region r values exceeded 0.50 in 8 of 13 regions, with the lowest values observed in cortex (r =+0.355) and anterior cingulate (r = +0.424). To establish whether the observed coordination is brain-specific, we performed a tissue-enrichment comparison against GTEx whole blood (n = 755) and 41 additional non-brain tissues. In whole blood, the mean pairwise PCDH correlation was r = +0.233 (99% positive pairs, 2,542/2,926 FDR-significant; Supplementary Figure S6), yielding a brain-to-blood ratio of 2.1:1 and establishing protocadherin coordination as a brain-enriched feature. The presence of positive PCDH co-expression in blood at roughly half the brain magnitude is consistent with low-level cross-tissue transcriptional co-regulation at the clustered PCDH locus.

**TABLE 1:** Within-region mean Pearson correlation of protocadherin gene pairs across human brain regions.

Region	n samples	n PCDH	n pairs	Mean r	95% CI	Median r	% positive
Nucleus accumbens	246	61	1,830	+0.595	+0.553 to +0.638	+0.623	99.7%
Putamen	205	47	1,081	+0.573	+0.525 to +0.621	+0.579	99.8%
Spinal cord	159	55	1,485	+0.558	+0.518 to +0.608	+0.602	100.0%
Cerebellar hemisphere	215	65	2,080	+0.557	+0.510 to +0.604	+0.592	99.7%
Hypothalamus	202	58	1,653	+0.539	+0.498 to +0.609	+0.548	100.0%
Caudate	246	59	1,711	+0.516	+0.477 to +0.566	+0.510	99.9%
Amygdala	152	53	1,378	+0.515	+0.467 to +0.580	+0.535	99.9%
Hippocampus	197	47	1,081	+0.508	+0.463 to +0.558	+0.520	99.8%
Substantia nigra	139	43	903	+0.458	+0.414 to +0.515	+0.453	99.6%
Cerebellum	241	65	2,080	+0.437	+0.383 to +0.490	+0.459	97.4%
Frontal cortex (BA9)	209	64	2,016	+0.430	+0.384 to +0.479	+0.452	94.2%
Anterior cingulate	176	60	1,770	+0.424	+0.375 to +0.484	+0.434	96.0%
Cortex	255	60	1,770	+0.355	+0.309 to +0.404	+0.350	94.5%

Within-region mean Pearson correlation coefficient (r) for all pairwise comparisons among expressed protocadherin family members (median TPM ≥ 1) in each of 13 GTEx brain regions, ranked by mean r. Sample size (n), number of expressed PCDHs, number of pairwise comparisons, mean r with 95% subject-level bootstrap confidence interval, median r, and the fraction of positive correlations are reported. All brain regions show predominantly positive within-region protocadherin co-expression.

### 3.2 Genome-wide screen identifies epigenetic regulators as top PCDH-coordinated genes in BA9

**FIGURE 2:**
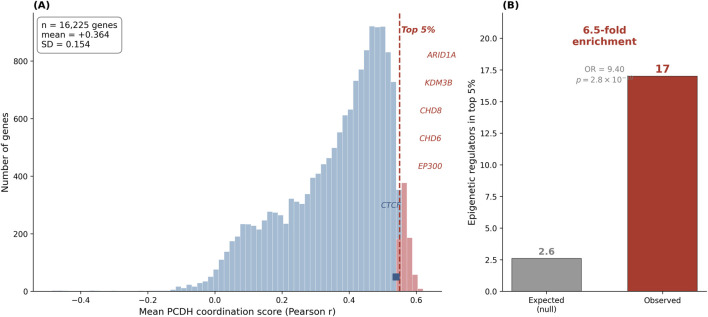
Genome-wide screen identifies preferential coordination of epigenetic regulators with the protocadherin family. **(A)** Distribution of mean PCDH coordination scores (Pearson r) for 16,225 brain-expressed genes in BA9 (frontal cortex), with the top 5% threshold (r ≥ 0.550) indicated. Selected epigenetic regulators (ARID1A, KDM3B, CHD8, CHD6, EP300) are labeled in red; CTCF is labeled in blue. **(B)** Expected (gray, 2.6) versus observed (red, 17) number of epigenetic regulators among the top 5% of PCDH-coordinated genes, corresponding to a 6.5-fold enrichment (Fisher’s exact OR = 9.40, p = 2.8 × 10^−10^).

The genome-wide co-expression screen in BA9 (n = 209) ranked all 16,225 brain-expressed genes by their mean Pearson correlation with the protocadherin family (Figure 2A). Mean PCDH-coordination scores ranged from −0.486 to +0.620 with a population mean of r = +0.364 (SD = 0.154). The top 5% threshold corresponded to r ≥ 0.550 (rank ≤ 811). Among the 52 brain-expressed epigenetic regulators in our curated set, 17 were observed in the top 5% of PCDH-coordinated genes compared to 2.6 expected under chance (Figure 2B), yielding 6.5-fold enrichment (Fisher’s exact OR = 9.40, p = 2.8 × 10^−10^).

**FIGURE 3:**
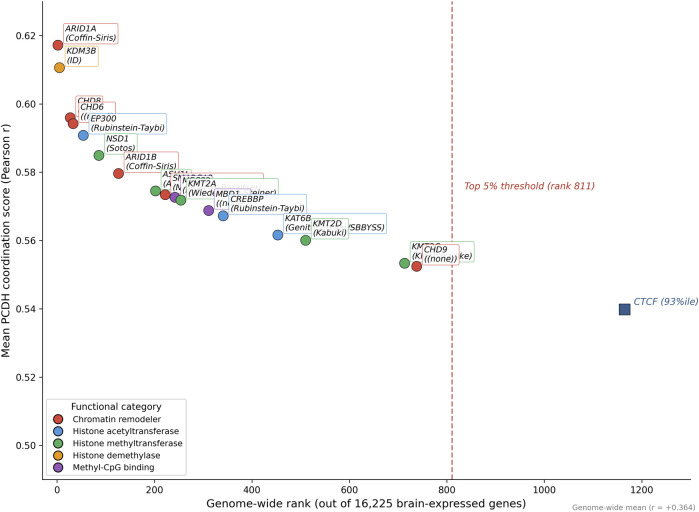
Top-ranked epigenetic regulators converge on chromatin-remodeling and histone-modifying enzymes implicated in neurodevelopmental syndromes. Scatter plot of mean PCDH coordination score versus genome-wide rank for brain-expressed genes in BA9 (n = 16,225). Colored dots indicate functional gene categories (chromatin remodeler, histone acetyltransferase, histone methyltransferase, histone demethylase, methyl-CpG binding); key syndrome-associated genes are labeled. The dashed vertical line marks the top 5% threshold at rank 811. CTCF (rank 1,165, r = 0.540) sits below the top-5% threshold but within the coordinated tail of the distribution.

Of the 17 epigenetic regulators in the top 5%, the highest-ranked were ARID1A (rank 2, r = 0.617), KDM3B (rank 5, r = 0.611), CHD8 (rank 27, r = 0.596), CHD6 (rank 33, r = 0.594), and EP300 (rank 54, r = 0.591) (Figure 3). CTCF, the canonical organizer of clustered PCDH locus looping, was at rank 1165 (r = 0.540; 92.8th percentile of the distribution), below the top-5% threshold but in the highly coordinated tail of the distribution. Cohesin complex members (RAD21, SMC1A, SMC3) ranked at the 81st, 80th, and 65th percentiles respectively. The pattern is consistent with the locus-organizing factors (CTCF, cohesin) being specifically tuned to the clustered PCDH locus while broader chromatin-remodeling activity covaries with protocadherin transcription across the brain.

Enrichment was robust to the choice of threshold cutoff. At the 1% threshold, fold-enrichment in BA9 was 13.5×; at 10%, 4.0×; with all regions remaining statistically significant at p < 0.001 (Supplementary Figure S4; Supplementary Table S3). Preranked gene set enrichment analysis confirmed threshold-free enrichment with a normalized enrichment score of NES = 2.27 in BA9 (FDR = 0.001) and NES = 1.85–3.23 across all four primary regions (Supplementary Figure S1; Supplementary Figure S2; Supplementary Table S4). The convergence of threshold-based and threshold-free analyses indicates the enrichment is not an artifact of the 5% cutoff.

### 3.3 Multi-region replication of enrichment

**FIGURE 4:**
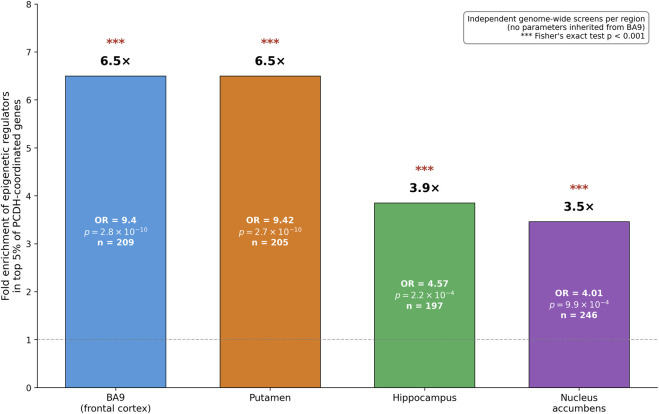
Regional replication of epigenetic-regulator enrichment across four independent brain regions. Fold enrichment of epigenetic regulators in the top 5% of PCDH-coordinated genes across BA9 frontal cortex (6.5×), Putamen (6.5×), Hippocampus (3.85×), and Nucleus accumbens (3.5×). Statistical significance for each region is indicated by three red asterisks; odds ratios, p-values, and sample sizes are shown within each colored bar. Independent genome-wide screens were run de novo in each region with no parameters inherited from the BA9 analysis (Fisher’s exact p < 0.001 in all regions).

The enrichment was independently confirmed in all four tested brain regions (Figure 4; Table 2). The genome-wide co-expression screen and Fisher’s exact test were run *de novo* in each region with no parameters inherited from the BA9 analysis. Enrichment magnitudes were 6.5× (BA9), 6.5× (putamen), 3.8× (hippocampus), and 3.5× (nucleus accumbens), with all p-values below 10^−3^. Epigenetic gene rankings were highly consistent across regions (pairwise Spearman ρ = 0.664−0.899, all p < 10^−7^; Supplementary Table S6). The five epigenetic genes with the lowest mean rank across all four regions were ARID1B (mean rank 67), SMARCA2 (81), ASH1L (105), ARID1A (142), and MECP2 (188) — all associated with neurodevelopmental syndromes. To assess whether the enrichment depends on inclusion of the broader PCDH family or is specific to canonical clustered protocadherins, the analysis was repeated restricted to the 28 clustered PCDHs (Supplementary Table S9). The clustered-only enrichment was 7.3-fold (OR = 11.17, p = 2.5 × 10^−12^) — modestly stronger than the full-family enrichment, confirming the result is not driven by non-clustered PCDH-related genes.

**TABLE 2:** Regional replication of epigenetic-regulator enrichment across four GTEx brain regions.

Region	n samples	Genes ranked	Epi in top 5%	Expected	Fold	OR (Fisher)	p-value
BA9 (frontal cortex)	209	16,225	17/52	2.6	6.5×	9.40	2.8 × 10⁻¹⁰
Putamen	205	15,020	17/52	2.6	6.5×	9.42	2.7 × 10⁻¹⁰
Hippocampus	197	15,274	10/52	2.6	3.85×	4.57	2.2 × 10⁻⁴
Nucleus accumbens	246	15,952	9/52	2.6	3.5×	4.01	9.9 × 10⁻⁴

Fold enrichment of brain-expressed epigenetic regulators (52-gene curated set expressed in BA9) in the top 5% of PCDH-coordinated genes across four GTEx brain regions. Sample size (n), total genes ranked, observed count of epigenetic regulators in the top 5%, expected count under chance, fold enrichment, Fisher's exact odds ratio, and p-value are reported. All four regions show statistically significant enrichment.

### 3.4 Robustness to cell-type composition and donor covariates

**FIGURE 5:**
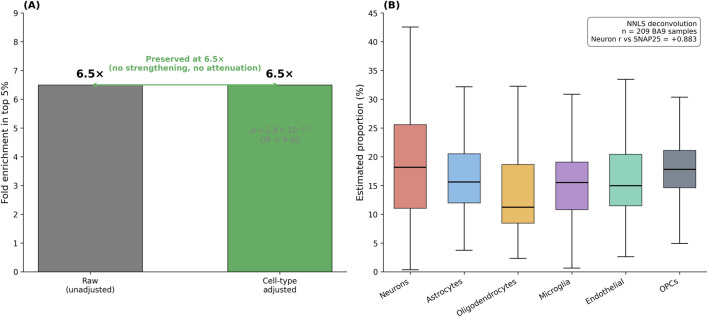
Cell-type adjustment preserves the epigenetic-regulator enrichment. **(A)** Raw fold enrichment of epigenetic regulators in the top 5% of PCDH-coordinated genes in BA9 (6.5×) compared to the cell-type-adjusted fold enrichment after partial-correlation adjustment for six estimated cell-type proportions (6.5×). Both yield Fisher’s exact OR = 9.40 and p = 2.8 × 10^−10^. The preservation of the enrichment under cell-type adjustment indicates that inter-individual variation in gross cell-type proportions does not account for the coordination signal; rank-order Spearman correlation between unadjusted and cell-type-adjusted gene rankings is ρ = 0.76—0.83. **(B)** Estimated cell-type proportions for six major brain cell types ‐ neurons, astrocytes, oligodendrocytes, microglia, endothelial cells, and oligodendrocyte precursor cells (OPCs) — across 209 BA9 samples, derived using non-negative least-squares (NNLS) deconvolution. Box plots illustrate inter-sample variability in cell-type composition.

NNLS-based cell-type deconvolution estimated mean BA9 proportions (n = 209): neurons 18.5% (SD = 10.4%), astrocytes 16.6% (SD = 7.1%), oligodendrocytes 14.6% (SD = 10.3%), microglia 15.9% (SD = 8.1%), endothelial cells 16.8% (SD = 6.9%), and OPCs 17.6% (SD = 5.7%) (Supplementary Figure S5). Estimated neuron proportions correlated strongly with established neuron markers: SYN1 (r = +0.749, p < 1 × 10^−50^), SNAP25 (r = +0.883, p < 1 × 10^−50^), and RBFOX3 (r = +0.780, p < 1 × 10^−50^), confirming biologically meaningful proportion estimates (Figure 5B; Table 3). After adjusting for cell-type proportions, the enrichment was preserved at 6.5-fold (Figure 5A) after partial-correlation adjustment for the six estimated cell-type proportions (OR = 9.40, p = 2.8 × 10^−10^), with rank-order Spearman ρ = 0.76−0.83 between unadjusted and cell-type-adjusted gene rankings. The preservation of the enrichment under cell-type adjustment indicates that inter-individual variation in gross cell-type proportions does not account for the observed coordination signal.

**TABLE 3:** Raw versus cell-type-adjusted epigenetic-regulator enrichment in BA9 frontal cortex.

Analysis	Epi in top 5%	Fold enrichment	OR	p-value
Raw (unadjusted)	17/52	6.5×	9.40	2.8 × 10⁻¹⁰
Cell-type adjusted	17/52	6.5×	9.40	2.8 × 10⁻¹⁰

Enrichment of epigenetic regulators in the top 5% of PCDH-coordinated genes in BA9 (n = 209), computed before and after partial-correlation adjustment for six NNLS-estimated cell-type proportions (neurons, astrocytes, oligodendrocytes, microglia, endothelial cells, OPCs). The enrichment is preserved at 6.5-fold after cell-type adjustment, indicating that gross cell-type composition does not account for the observed coordination signal.

Adjustment for donor age, sex, and RIN preserved the enrichment (Supplementary Table S10): 7.7-fold, OR = 12.14, p = 1.6 × 10^−13^. Stratification of BA9 samples by RIN quartile revealed substantively consistent PCDH co-expression across quartiles (Supplementary Figure S3), with no monotonic decline in mean within-region correlation as RIN decreased. The result is therefore not explained by demographic or RNA-quality covariation.

### 3.5 Convergence on neurodevelopmental disorder genes

The top-ranked epigenetic regulators converge on a set of genes associated with well-characterized neurodevelopmental syndromes (Figure 3): ARID1A and ARID1B (Coffin-Siris syndrome), CREBBP and EP300 (Rubinstein-Taybi syndrome), KMT2D (Kabuki syndrome), NSD1 (Sotos syndrome), MECP2 (Rett syndrome), KMT2A (Wiedemann-Steiner syndrome), SMARCA2 (Nicolaides-Baraitser syndrome), KDM5C (X-linked intellectual disability), CHD8 (autism spectrum disorder), and ASH1L (autism spectrum disorder). The convergence is consistent with shared transcriptional programs linking chromatin-remodeling capacity to protocadherin-dependent neuronal identity, offering a candidate axis through which broad chromatin-remodeling perturbations may propagate to PCDH-dependent neurodevelopmental phenotypes.

### 3.6 Cross-dataset replication and preservation across conditions

**FIGURE 6:**
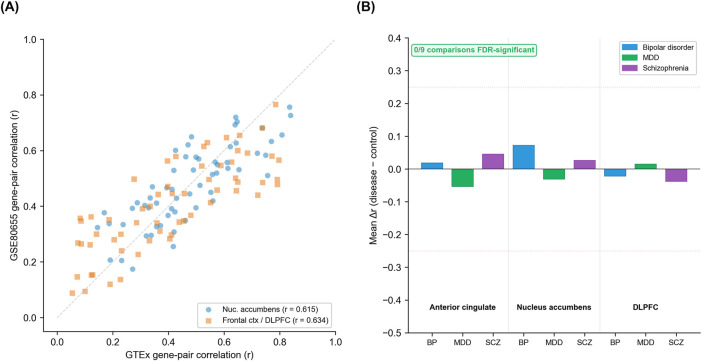
Independent-dataset replication and preservation of protocadherin co-expression across psychiatric conditions. **(A)** Scatter plot comparing gene-pair Pearson correlation coefficients between the GTEx and GSE80655 datasets for nucleus accumbens (blue circles, cross-dataset r = 0.615) and frontal cortex / DLPFC (orange squares, cross-dataset r = 0.634). Each set of points closely tracks the diagonal, indicating strong cross-dataset agreement. **(B)** Grouped bar chart of mean correlation differences (disease minus control) across anterior cingulate, nucleus accumbens, and DLPFC for bipolar disorder, major depressive disorder, and schizophrenia. All differences are close to zero; no FDR-significant differences are observed across the nine condition‐region comparisons.

Protocadherin co-expression patterns were independently replicated in GSE80655 (Figure 6A; Table 4). Within-region control correlations in GSE80655 were anterior cingulate r = +0.594 (2,331/2,775 pairs FDR-significant), nucleus accumbens r = +0.569 (2,066/2,775), and DLPFC r = +0.576 (2,167/2,775). Cross-dataset validation of individual gene-pair effect sizes yielded high consistency: anterior cingulate r = 0.629 (p < 1 × 10^−50^), nucleus accumbens r = 0.615 (p < 1 × 10^−50^), and DLPFC vs. BA9 r = 0.634 (p < 1 × 10^−50^). The patterns observed in GTEx are therefore not specific to GTEx; they replicate in an independent brain-bank cohort.

**TABLE 4:** Cross-dataset replication of protocadherin co-expression in the GSE80655 brain bank.

Region	n controls	Mean r (within)	FDR-significant pairs	Cross-dataset r
Anterior cingulate	24	+0.594	2,331/2,775	0.629
Nucleus accumbens	22	+0.569	2,066/2,775	0.615
DLPFC	24	+0.576	2,167/2,775	0.634

Within-region mean Pearson correlation (r) of expressed protocadherin gene pairs in GSE80655 control samples for anterior cingulate, nucleus accumbens, and DLPFC, the number of FDR-significant pairs (Benjamini–Hochberg, α = 0.05), and the cross-dataset Pearson r between GTEx-derived and GSE80655-derived gene-pair correlations within each region.

In GSE80655, no FDR-significant disruptions of protocadherin co-expression were detected across bipolar disorder, major depressive disorder, or schizophrenia in any of the three brain regions tested (nine total comparisons; Figure 6B). Mean change in correlation (Δr) ranged from −0.085 to +0.103 across conditions and regions, with 62%–91% of protocadherin pairs showing preservation at the >80% level across the nine condition‐region cells. Protocadherin co-expression is preserved in psychiatric disease, consistent with a constitutive transcriptional feature rather than a state-dependent one. Methylation-level analysis in GSE131706 BA9 prefrontal cortex (n = 32) identified coordinated methylation patterns at CpG probes mapping to clustered PCDH promoters and DNMT genes, providing a complementary epigenetic signal at the methylation layer that mirrors the transcriptional coordination observed at the RNA layer (Supplementary Table S2).

### 3.7 Negative findings

We report two negative findings transparently. First, the SIGMAR1 co-expression analysis we conducted in parallel did not produce comparable enrichment; SIGMAR1 ranked at the 77th percentile of the PCDH-coordination distribution and was not in the top 5% of coordinated genes. Second, an attempted replication in a smaller independent neurodegenerative brain bank cohort produced quantitatively weaker enrichment, which we attribute to the smaller sample size and the disease-associated variation rather than to a failure of the primary finding (full discussion in Supplementary Note 1).

## 4 Discussion

This study identifies a statistically robust co-expression association between epigenetic regulatory genes and protocadherin family members across the human brain, present in all four independently analyzed brain regions, confirmed by threshold-free GSEA, and surviving cell-type deconvolution and multiple sensitivity analyses. All findings are correlational; the analyses are designed to generate testable hypotheses rather than to establish mechanistic relationships or causal directionality.

The 6.5-fold enrichment of epigenetic regulators in the top 5% of PCDH-coordinated genes (OR = 9.40, p = 2.8 × 10^−10^) was independently replicated across four brain regions (fold enrichments 3.5× to 6.5×, p-values 9.9 × 10^−4^ to 2.8 × 10^−10^). The consistency of individual gene rankings across regions (Spearman rho = 0.664 to 0.899) indicates that the same epigenetic regulators dominate the PCDH co-expression hierarchy regardless of brain region, suggesting a general feature of human brain gene regulation rather than a region-specific phenomenon.

The threshold sensitivity analyses substantially strengthen this conclusion. Enrichment at the most stringent threshold (top 1%) is stronger than at 5% or 10% in all four regions (9.6–13.5×), indicating that the highest-coordinated genes are disproportionately enriched for epigenetic regulators. This pattern is precisely the opposite of what would be expected from a false positive or threshold artifact. Preranked GSEA (NES = 1.85–3.23, all FDR = 0.001) independently confirmed this in a framework that does not use any threshold, evaluating the complete distribution of gene ranks.

The cell-type deconvolution analysis addresses the principal methodological concern for bulk RNA-seq co-expression analysis: whether observed correlations reflect within-cell gene co-expression or merely inter-individual variation in cell-type proportions. After partialing out estimated proportions of six major cortical cell types, the enrichment of epigenetic regulators persisted unchanged at 6.5-fold (OR 9.40, p = 2.8 × 10^−10^), with rank-order Spearman rho = 0.76 to 0.83 between unadjusted and cell-type-adjusted gene rankings. This preservation indicates that gross cell-type composition does not account for the observed coordination signal. While reference-based deconvolution from bulk data cannot substitute for single-cell validation, and residual confounding by cell-state heterogeneity within cell types cannot be fully excluded, these results indicate that gross cell-type composition alone cannot account for the observed co-expression pattern. Single-cell RNA-seq profiling of matched brain regions, or spatial transcriptomic approaches (e.g., MERFISH, 10x Visium) with cell-type resolution, would be required to determine whether the co-expression relationship is cell-intrinsic.

The co-expression patterns observed here are consistent with the established biology of protocadherin locus regulation. The clustered PCDH locus requires coordinated chromatin remodeling, histone modification, and CTCF-mediated looping for appropriate promoter selection (Guo et al., 2012; Monahan et al., 2012; Canzio et al., 2019). The co-expression data extend this picture to show that, at the population level—across hundreds of individuals—variation in the expression of these regulatory genes tracks with variation in protocadherin expression. Whether this co-expression reflects direct co-regulation of PCDH loci by the identified chromatin regulators, shared upstream transcriptional programs, or other mechanistic explanations cannot be determined from co-expression data alone. Experimental approaches including CRISPRi/dCas9-based epigenetic perturbation in iPSC-derived neurons and single-cell analyses of matched samples would be required to test mechanistic hypotheses directly.

The convergence of neurodevelopmental disorder genes on protocadherin co-expression is a biologically notable observation that warrants careful framing. Coffin-Siris syndrome (ARID1A/ARID1B), Rubinstein-Taybi syndrome (EP300/CREBBP), Kabuki syndrome (KMT2D), Sotos syndrome (NSD1), Rett syndrome (MECP2), and autism spectrum disorder (CHD8, ASH1L) are caused by mutations in genes with diverse enzymatic activities that converge on overlapping neurodevelopmental phenotypes (Tsurusaki et al., 2012; Roelfsema et al., 2005; Bernier et al., 2014; Amir et al., 1999; Ng et al., 2010; Tatton-Brown et al., 2014; Jones et al., 2012; Kurotaki et al., 2002). The observation that these same genes rank among the strongest PCDH co-expression partners in healthy human brain, consistently across four brain regions, is consistent with the hypothesis that altered PCDH co-expression may represent one downstream correlate of epigenetic regulator dysfunction in neurodevelopmental conditions. This is a hypothesis, not a conclusion: the present co-expression data do not establish that mutation of any of these genes causes PCDH network disruption, nor do they demonstrate that such disruption contributes to the clinical phenotype. Direct testing in disease-relevant model systems—brain organoids from individuals with NDD-associated mutations, or iPSC-derived neurons with isogenic epigenetic perturbations—will be required to evaluate this hypothesis.

The RNA quality stratification analysis addresses the observation that controls from neurodegenerative disease brain banks show markedly reduced PCDH co-expression relative to GTEx and GSE80655. The finding that PCDH co-expression is flat across the full range of GTEx RIN values (Δ = −0.057 from Q1 to Q4) demonstrates that RNA degradation at the levels present in GTEx does not suppress the co-expression signal. The brain bank discrepancy therefore likely reflects biological differences between cohorts—including subclinical neuropathological burden, altered cell-type compositions secondary to protracted disease courses, or agonal state effects—rather than technical RNA quality differences. This interpretation is consistent with documented differences in transcriptomic profiles between general-population brain banks and disease-bank controls (Hawrylycz et al., 2012; Zhang et al., 2016). Future studies using control cohorts from general-population brain banks alongside matched neurodegenerative cases, with neuropathological staging of control specimens, will be essential to resolve this observation.

The complete preservation of protocadherin coordination across psychiatric conditions (bipolar disorder, MDD, schizophrenia) is consistent with the understanding that these conditions involve altered neural circuit function rather than substantial neuronal loss or disruption of basic neuronal identity machinery (Gandal et al., 2018). This observation suggests that protocadherin co-expression may be preferentially altered by conditions involving more severe cellular pathology. The methylation-level coupling observed in ASD brain tissue (GSE131706) parallels this expression-level preservation and suggests that epigenetic-PCDH coupling may be fundamentally robust, though replication in neurotypical control methylation data is needed (Wong et al., 2019; Ramaswami et al., 2020).

The co-expression framework presented here complements emerging computational approaches in regulatory genomics. Deep learning-based methods for epigenetic modification prediction (e.g., EnDeep4mC; Zhang et al., 2026), graph-based regulatory network modeling (e.g., HiGLDP; Wang et al., 2026, MHHGRN; Wu et al., 2024), and spatial transcriptomics integration frameworks (e.g., MMSpa; Liu et al., 2026) offer complementary perspectives on the regulatory landscape. The co-expression approach employed here is distinguished by its use of large-scale population-level RNA-seq data (n = 2,642) and its focus on detecting genome-wide coordination patterns rather than predicting individual regulatory interactions. Future integration of these approaches—for example, using GSEA rankings to prioritize targets for deep learning-based regulatory modeling, or applying spatial transcriptomics to validate spatially resolved co-expression signatures—represents a productive direction for the field.

### 4.1 Limitations

First, all primary analyses used bulk RNA-seq data, which averages expression across multiple cell types within each tissue sample. While cell-type deconvolution demonstrated that the enrichment is preserved after adjustment for estimated cell-type proportions, reference-based NNLS deconvolution provides approximate relative estimates. Residual confounding by cell-type composition or cell-state variation within cell types cannot be fully excluded from bulk RNA-seq data. The co-expression relationships observed here may partly reflect shared developmental cell-type identity programs rather than purely cell-intrinsic regulatory relationships. Single-cell RNA-seq validation would be required for definitive resolution.

Second, co-expression is a correlational measure and does not establish causal or mechanistic relationships. All findings are interpreted as hypothesis-generating. The enrichment of epigenetic regulators among PCDH-coordinated genes is consistent with known biology of PCDH locus regulation but does not demonstrate that variation in epigenetic gene expression directly affects protocadherin expression levels.

Third, the curated list of 54 epigenetic regulators, though drawn from published literature across five functional categories, may not be exhaustive. The threshold sweep and GSEA provide evidence that enrichment is not dependent on precise gene set boundaries.

Fourth, the methylation-level analysis (GSE131706) was conducted in brain tissue from individuals with autism spectrum disorder (n = 32) rather than neurotypical controls. The small sample size and disease-specific population limit generalizability; replication in a control methylation dataset would strengthen this line of evidence.

Fifth, the GTEx cohort consists of post-mortem tissue from deceased adult donors, which may not fully represent the general population, and co-expression patterns during development (when protocadherin isoform selection is actively regulated) may differ from adult patterns.

### 4.2 Concluding remarks

Inter-individual variation in protocadherin gene expression across the human brain is preferentially co-expressed with variation in epigenetic regulatory gene expression. This association replicated across four brain regions, survived threshold sensitivity analyses (including threshold-free GSEA), cell-type proportion adjustment, independent dataset replication, and donor covariate adjustment. Bootstrap confidence intervals confirmed positive co-expression in all 13 GTEx brain regions, with all 95% CIs above zero. RIN stratification demonstrated that RNA quality alone does not explain brain bank co-expression discrepancies, pointing toward biological rather than technical explanations. The convergence of neurodevelopmental disorder genes at the top of the PCDH co-expression ranking, consistently across brain regions, is consistent with a hypothesis linking epigenetic regulatory networks to protocadherin expression that merits experimental investigation. These findings provide a transcriptomic framework for future mechanistic studies and generate testable hypotheses about the shared downstream consequences of genetically distinct neurodevelopmental disorder mutations.

## Data Availability

The original contributions presented in the study are publicly available. These data can be found in the GTEx v8 repository under dbGaP accession number phs000424.v8.p2 (https://gtexportal.org), and in the NCBI Gene Expression Omnibus (GEO) repository under accession numbers GSE80655 (https://www.ncbi.nlm.nih.gov/geo/query/acc.cgi?acc=GSE80655) and GSE131706 (https://www.ncbi.nlm.nih.gov/geo/query/acc.cgi?acc=GSE131706). Analysis code and intermediate results are publicly available in the GitHub repository at https://github.com/nwharbert8-ui/epigenetic-pcdh-coordination.

## Supplementary material

SUPPLEMENTARY FIGURE S1 GSEA enrichment plot for epigenetic regulators in BA9 frontal cortex. Gene set enrichment analysis (GSEA) plot for the 52-gene curated epigenetic regulator set against the genome-wide PCDH-coordination ranking in BA9 (n = 209). Normalized enrichment score NES =2.27, nominal p < 0.001, FDR < 0.001 from 1,000 permutations.

SUPPLEMENTARY FIGURE S2 GSEA enrichment plots across three additional brain regions. GSEA plots for the 52-gene epigenetic regulator set against the genome-wide PCDH-coordination rankings in Putamen (NES = 3.23), Hippocampus (NES = 1.85), and Nucleus accumbens (NES = 1.95). All FDR < 0.001.

SUPPLEMENTARY FIGURE S3 RNA integrity stratification — protocadherin co-expression is not driven by RNA quality. Within-region mean PCDH r across BA9 samples stratified by RNA Integrity Number (RIN) quartile. The strong positive co-expression pattern is preserved across RIN quartiles (Δ = ‐0.057 from Q1 to Q4), indicating the signal is not an artifact of degraded RNA samples.

SUPPLEMENTARY FIGURE S4 Threshold-sweep visualization for the epigenetic-regulator enrichment. Fold enrichment of epigenetic regulators in the top X% of PCDH-coordinated genes, with X swept from 1% to 25% across the four primary brain regions. Enrichment is monotonically stronger at more stringent thresholds and remains statistically significant across the tested range.

SUPPLEMENTARY FIGURE S5 Cell-type proportion distributions across BA9 samples. Distributions of NNLS-estimated cell-type proportions across all 209 BA9 samples used in the cell-type-adjusted analysis. Distributions are consistent with the published biology of adult human prefrontal cortex composition.

SUPPLEMENTARY FIGURE S6 Tissue-enrichment specificity — PCDH co-expression is brain-enriched. Tissue-specificity comparison of within-region PCDH co-expression across 13 GTEx brain regions versus whole blood and 41 non-brain tissues, demonstrating the brain-enrichment of the coordinated protocadherin co-expression signal.

SUPPLEMENTARY NOTE 1 SIGMAR1 negative-finding analysis. Targeted analysis testing whether SIGMAR1 — a chaperone receptor of independent research interest — shows the same preferential PCDH coordination as the broader epigenetic-regulator set. SIGMAR1’s rank in the PCDH-coordination distribution (rank 3,670, 77th percentile, outside the top 5%) and its mean PCDH-coordination r value are reported.

SUPPLEMENTARY TABLE S1 Full genome-wide PCDH-coordination rankings (BA9). All 16,225 brain-expressed genes (median TPM ≥ 1) in BA9 frontal cortex (n = 209) ranked by their mean Pearson correlation with the 77 protocadherin family members. Columns: gene symbol, PCDH-coordination score, rank, percentile, epigenetic regulator membership flag, protocadherin family membership flag.

SUPPLEMENTARY TABLE S2 Curated epigenetic regulator panel (54 genes). The 54-gene curated epigenetic regulator panel used in the enrichment analysis, including functional category (chromatin remodeler, histone acetyltransferase, histone methyltransferase, histone demethylase, methyl-CpG binding, DNA methyltransferase), associated neurodevelopmental syndrome, BA9 rank in the genome-wide screen, and BA9 PCDH-coordination score.

SUPPLEMENTARY TABLE S3 Threshold-sweep statistics for epigenetic-regulator enrichment. Fold enrichment, Fisher’s exact odds ratio, and p-value for the 52-gene epigenetic regulator panel against the top 1%, 5%, and 10% of PCDH-coordinated genes across all four primary brain regions.

SUPPLEMENTARY TABLE S4 GSEA results for the epigenetic regulator panel. Preranked GSEA statistics (enrichment score ES, normalized enrichment score NES, nominal p-value, FDR) for the 52-gene epigenetic regulator set against the genome-wide PCDH-coordination ranking in each of the four primary brain regions.

SUPPLEMENTARY TABLE S5 Multi-region enrichment details. Extended multi-region enrichment statistics: per-region sample size, number of expressed PCDHs and epigenetic regulators included, top-5% cutoff in r and rank space, observed and expected counts, fold enrichment, Fisher’s exact OR with confidence interval, and p-value.

SUPPLEMENTARY TABLE S6 Cross-region consistency of epigenetic-regulator rankings. Pairwise Spearman rank correlations of epigenetic regulator PCDH-coordination scores across the four primary brain regions, with p-values. All ρ values 0.664‐0.899, all p < 10^−7^.

SUPPLEMENTARY TABLE S7 Cell-type deconvolution and adjusted PCDH-coordination rankings (BA9). Per-sample NNLS-estimated proportions of six brain cell types across 209 BA9 samples, alongside the cell-type-adjusted PCDH-coordination scores obtained by partial correlation regressing out the six cell-type proportion variables. Adjusted rankings and percentiles are provided for all 16,225 brain-expressed genes.

SUPPLEMENTARY TABLE S8 GSE80655 cross-dataset replication details. Per-region within-cohort PCDH co-expression statistics in the GSE80655 brain bank (anterior cingulate, nucleus

SUPPLEMENTARY TABLE S9 Sensitivity analyses. Sensitivity-analysis statistics including the clustered-only PCDH analysis (28 clustered PCDHs: 7.3-fold, OR = 11.17, p = 2.5 × 10^⁻12^), covariate-adjusted enrichment (6.5-fold), and combined clustered + covariate-adjusted enrichment (7.7-fold, OR = 12.14, p = 2.1 × 10^⁻13^).

SUPPLEMENTARY TABLE S10 Covariate-adjusted PCDH-coordination rankings (BA9). BA9 PCDH-coordination scores after linear-regression residualization of expression values against donor age, sex, and RIN, with the corresponding 16,225-gene ranking and the post-adjustment enrichment statistics for the 52-gene epigenetic regulator panel.
