# Supplementary figures and images for: Epigenetic regulators are preferentially coordinated with protocadherin gene expression across the human brain: a genome-wide co-expression analysis
Source: Front Genet. 2026 Jul 9;17:1807347. doi: 10.3389/fgene.2026.1807347 (PMC13391046; doi:10.3389/fgene.2026.1807347)

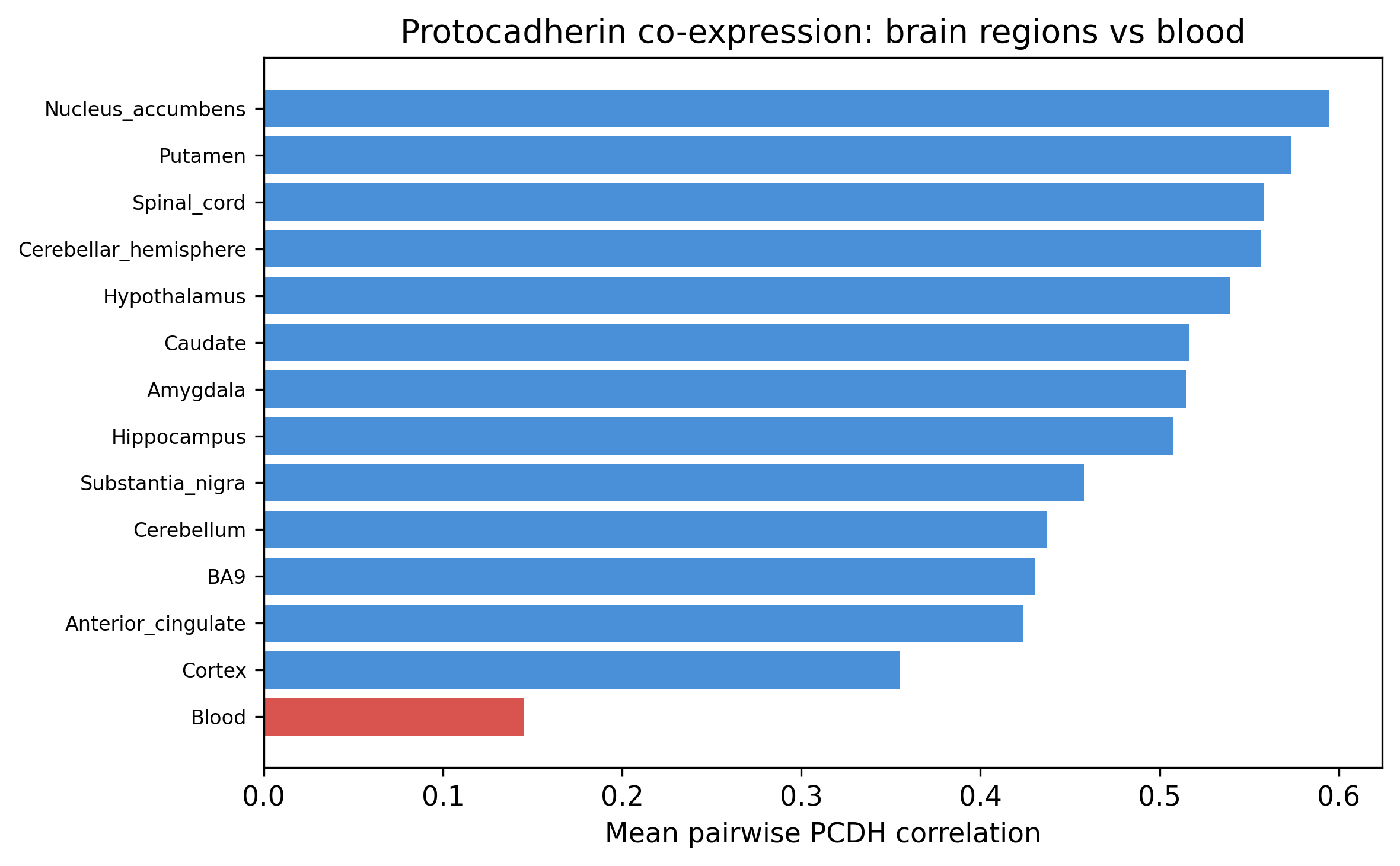

Supplement: Supplementary file 1 [file Image5.png]

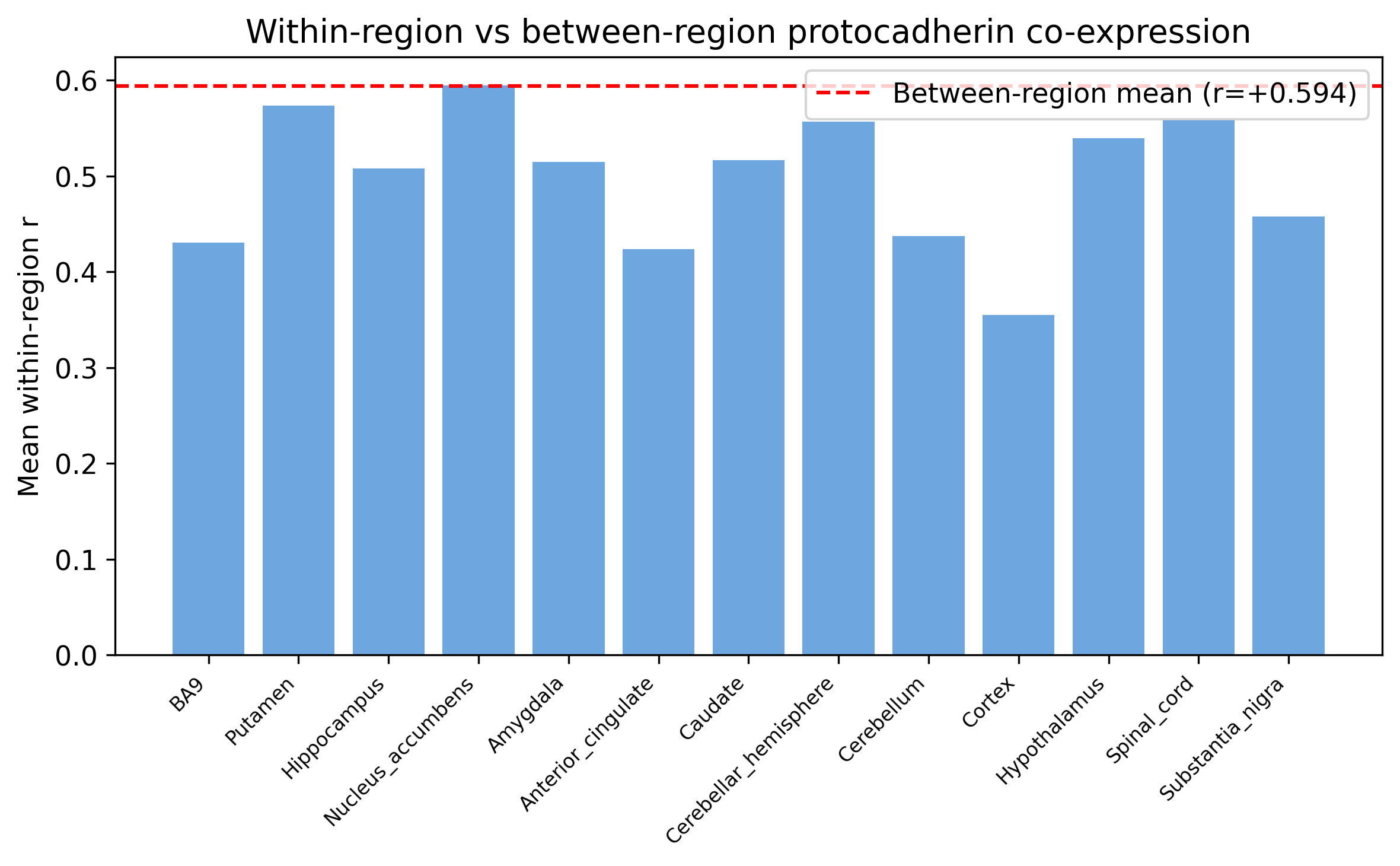

Supplement: Supplementary file 2 [file Image4.png]

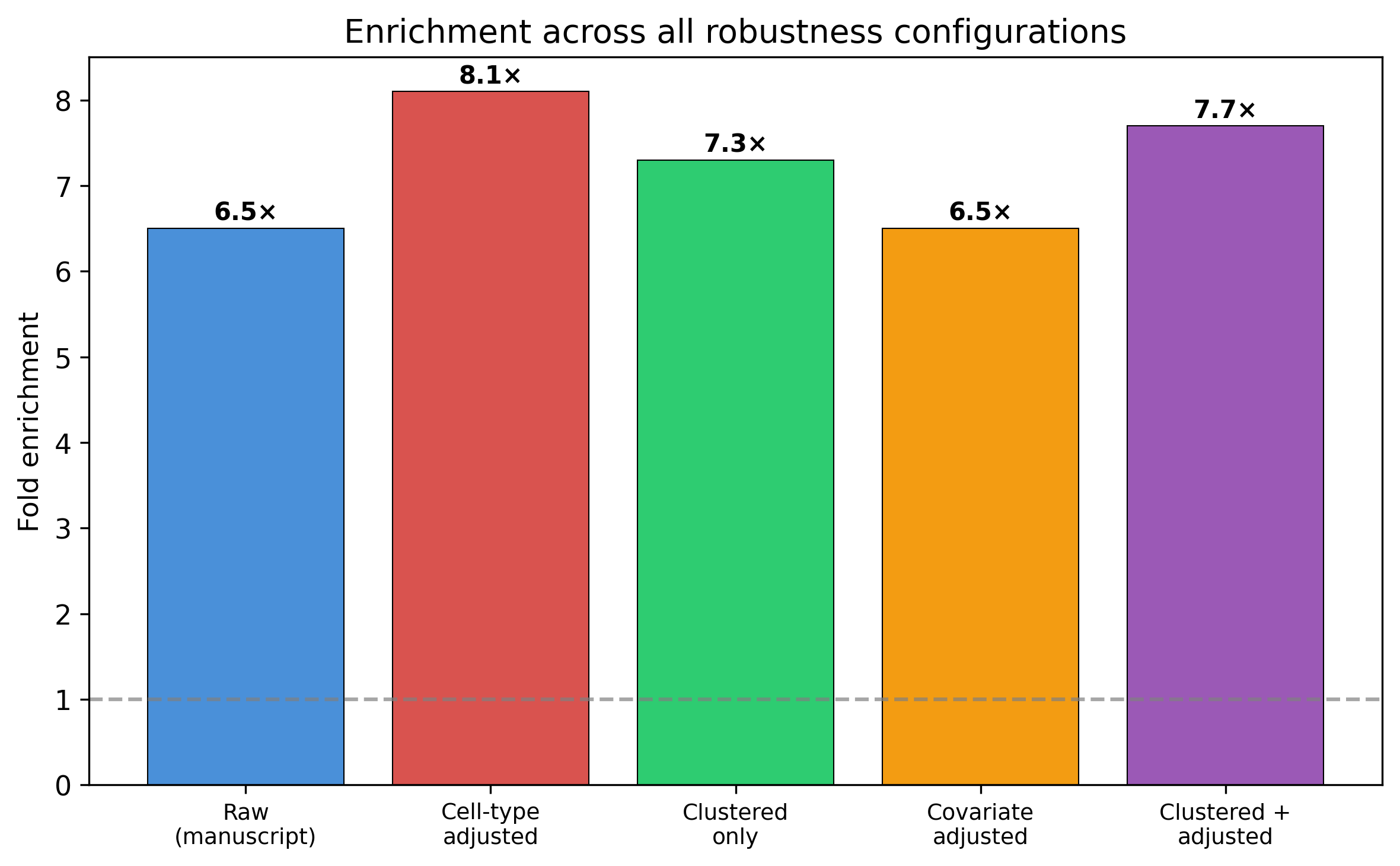

Supplement: Supplementary file 3 [file Image2.png]

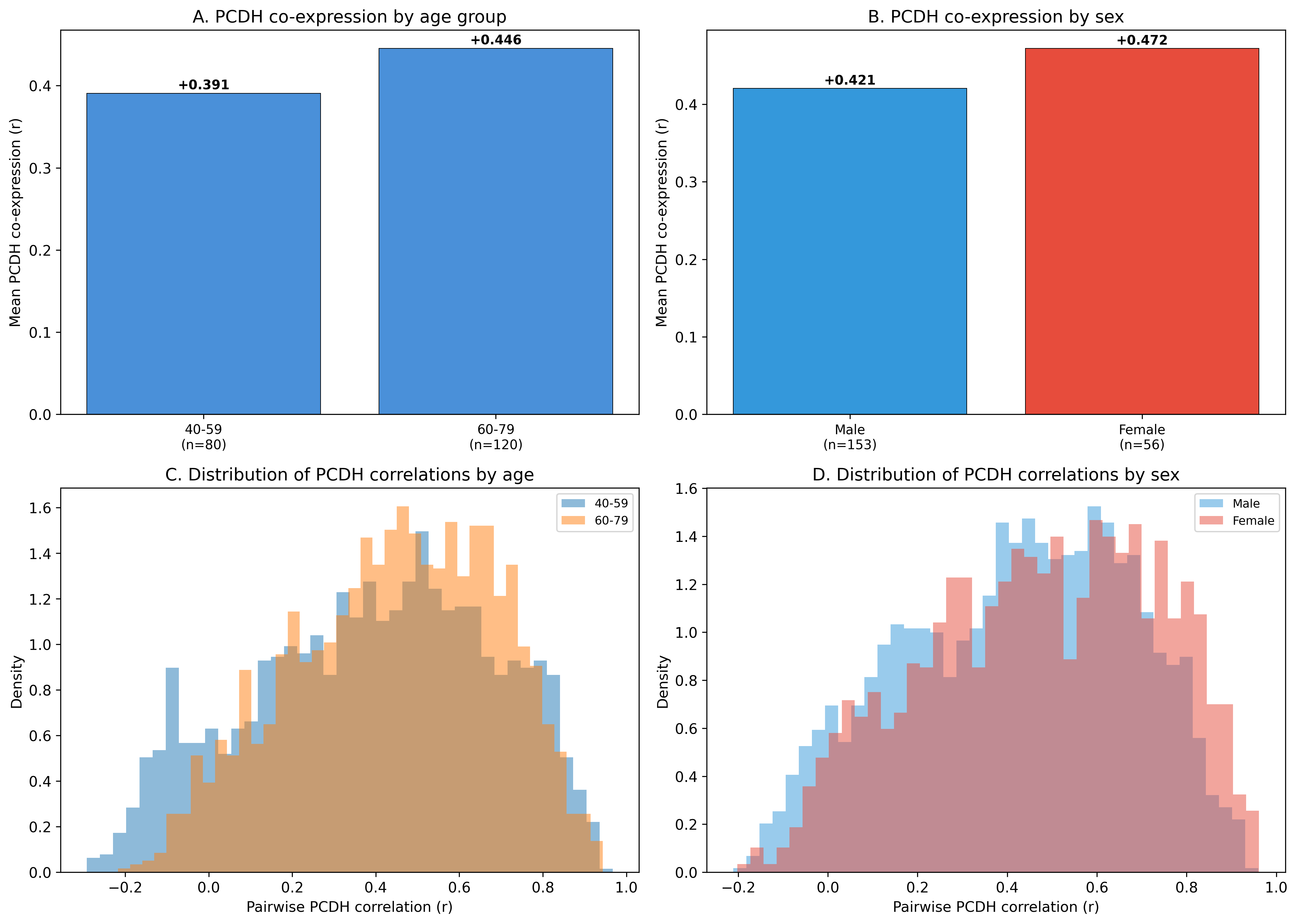

Supplement: Supplementary file 4 [file Image1.png]

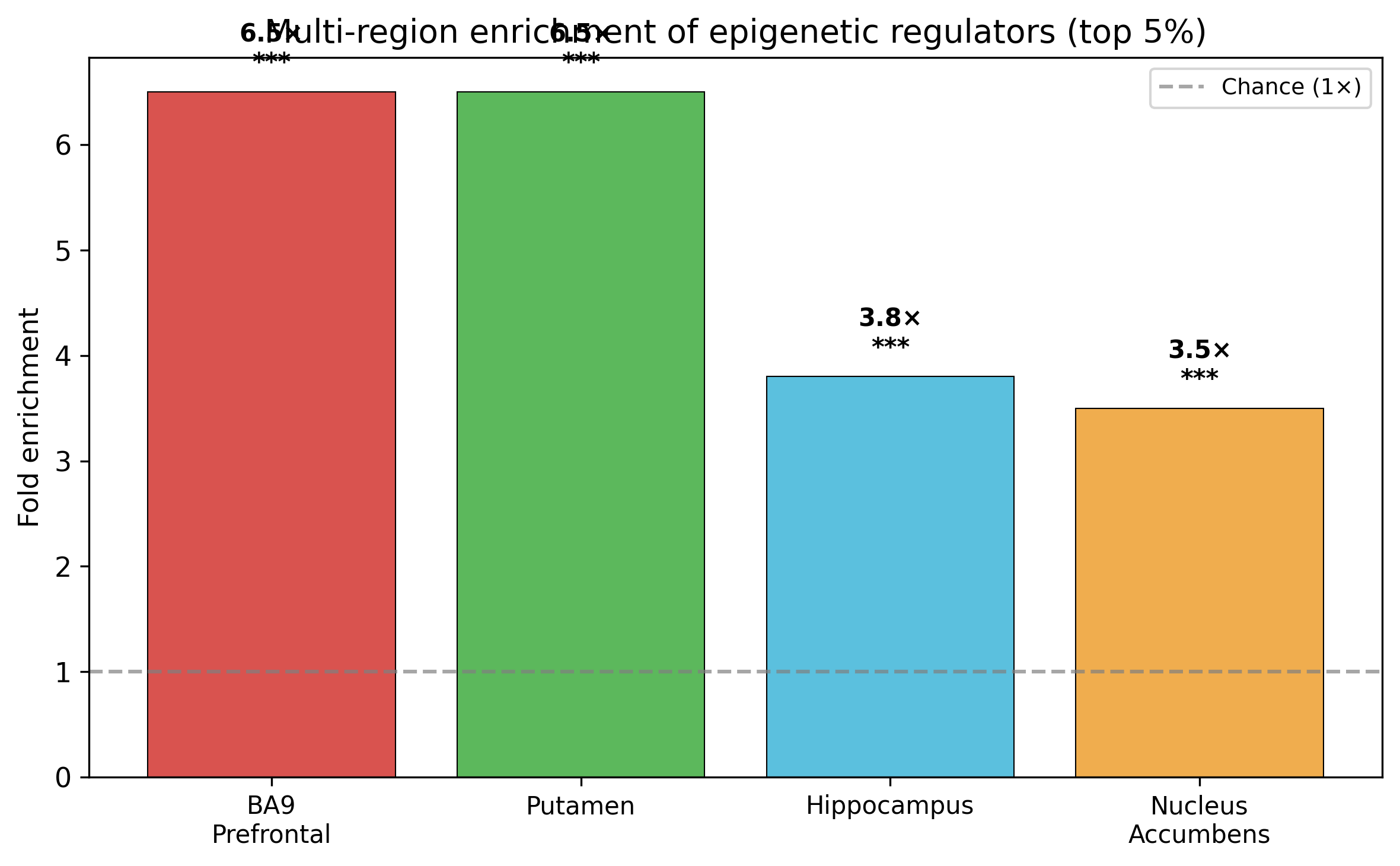

Supplement: Supplementary file 5 [file Image3.png]
